# Characterization and Comparison of Contrast Imaging Properties of Naturally Isolated and Heterologously Expressed Gas Vesicles

**DOI:** 10.3390/ph17060755

**Published:** 2024-06-07

**Authors:** Tingting Liu, Jieqiong Wang, Chenxing Liu, Yuanyuan Wang, Zhenzhou Li, Fei Yan

**Affiliations:** 1 Ultrasonic Medicine, Graduate School, Guangxi University of Chinese Medicine, Nanning 530200, China; ltingting949@gmail.com; 2 Department of Ultrasound, The Second People’s Hospital of Shenzhen, The First Affiliated Hospital of Shenzhen University, Shenzhen 518061, China; 3 Department of Rehabilitation Medicine, Huashan Hospital, Fudan University, Shanghai 201206, China; jq.wang@siat.ac.cn; 4 Center for Cell and Gene Circuit Design, CAS Key Laboratory of Quantitative Engineering Biology, Shenzhen Institute of Synthetic Biology, Shenzhen Institutes of Advanced Technology, Chinese Academy of Sciences, Shenzhen 518055, China; cx.liu2@siat.ac.cn (C.L.); yy.wang7@siat.ac.cn (Y.W.)

**Keywords:** nanoscale contrast agents, gas vesicles, genetic engineering, bio-synthesize, ultrasound imaging

## Abstract

Nanoscale ultrasound contrast agents have attracted considerable interest in the medical imaging field for their ability to penetrate tumor vasculature and enable targeted imaging of cancer cells by attaching to tumor-specific ligands. Despite their potential, traditional chemically synthesized contrast agents face challenges related to complex synthesis, poor biocompatibility, and inconsistent imaging due to non-uniform particle sizes. To address these limitations, bio-synthesized nanoscale ultrasound contrast agents have been proposed as a viable alternative, offering advantages such as enhanced biocompatibility, consistent particle size for reliable imaging, and the potential for precise functionalization to improve tumor targeting. In this study, we successfully isolated cylindrical gas vesicles (GVs) from *Serratia. 39006* and subsequently introduced the GVs-encoding gene cluster into *Escherichia coli* using genetic engineering techniques. We then characterized the contrast imaging properties of two kinds of purified GVs, using in vitro and in vivo methods. Our results demonstrated that naturally isolated GVs could produce stable ultrasound contrast signals in murine livers and tumors using clinical diagnostic ultrasound equipment. Additionally, heterologously expressed GVs from gene-engineered bacteria also exhibited good ultrasound contrast performance. Thus, our study presents favorable support for the application of genetic engineering techniques in the modification of gas vesicles for future biomedical practice.

## 1. Introduction

Nanoscale ultrasound contrast agents have garnered significant attention in the field of medical imaging due to their ability to penetrate through the vasculature into adjacent tumor tissues [1]. This unique characteristic greatly expands their applications in disease diagnosis and therapy, especially in tumors [2,3]. Traditional nanoscale contrast agents are mostly chemically synthesized and have limitations in terms of complex preparation processes, poor biocompatibility, and non-uniform particle sizes [4,5]. To overcome these limitations, there has been a growing interest in the development of bio-synthesized nanoscale ultrasound contrast agents. These bio-synthesized nanobubbles offer several advantages over their chemically synthesized counterparts. Firstly, they exhibit excellent biocompatibility and low toxicity, ensuring minimal harm to the body [6]. Secondly, their uniform particle sizes ensure consistent and reliable ultrasound signal enhancement, resulting in accurate and high-quality imaging [7,8,9]. Additionally, bio-synthesized contrast agents can be easily modified or functionalized to enhance their targeting ability, enabling personalized and precise tumor diagnosis [10,11]. Importantly, bio-synthesized ultrasound contrast agents have an approximately 100–200 nm particle size, making it possible for them to traverse through the tumor vessel via the enhanced permeation and retention (EPR) effect [12]. By modifying these bio-synthesized nanobubbles with ligands that target tumor cell markers or receptors, they can specifically bind onto the tumor cells. This greatly facilitates the early detection, staging, and monitoring of tumors [13,14,15].

To date, gas vesicles have been successfully extracted from *Halobacteria NRC-1*, and their ultrasound contrast imaging performance has been examined in mouse liver and tumors using clinical ultrasound diagnostic equipment [16]. In this study, we isolated and characterized gas vesicles (GV_Ser_) from *Serratia. 39006*, and, importantly, we cloned the gene cluster-encoding gas vesicles from *Serratia. 39006* and inserted them into the pET-28a vector. The heterologous expression of gas vesicles (GV_E.coli_) was achieved in *E. coli* BL21 (A1). The physical and chemical properties of gas vesicles from *Serratia. 39006* and gas vesicles from *E. coli* BL21 (A1) were characterized, and their ultrasound contrast imaging performance was determined in vitro and in vivo.

## 2. Results

### 2.1. Characterization of GVs from Serratia and 39006 E. coli

**Figure 1:**
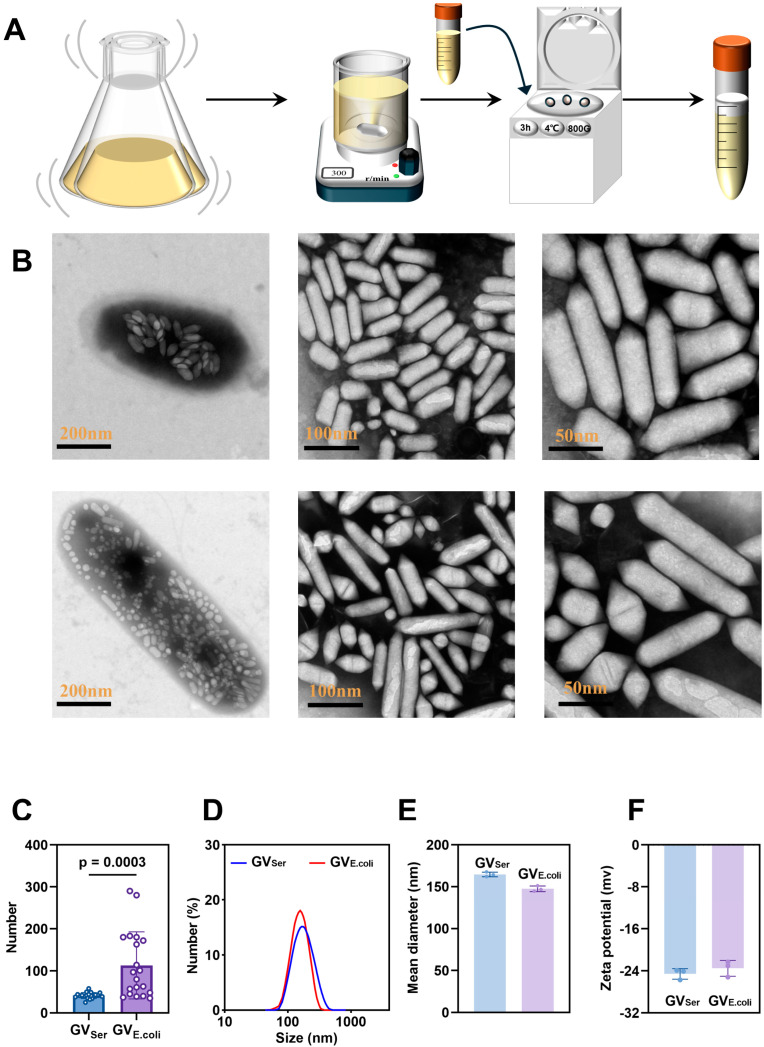
Preparation and characterization of GVs. (**A**) Diagram of preparation of biosynthetic GVs; (**B**) TEM images of *Serratia* bacteria and GV_Ser_ (top row) and *39006 E. coli* bacteria and GV_E.coli_ (bottom row), with scale bars indicated from left to right as 200 nm, 100 nm, and 50 nm. (**C**) The number of GVs within each bacterium, as shown under the TEM, n = 20. (**D**) Particle size distributions of GV_Ser_ and GV_E.coli_. (**E**) Mean diameters of GV_Ser_ and GV_E.coli_. (**F**) Zeta potentials of GV_Ser_ and GV_E.coli_.

The GVs were extracted from *Serratia* and genetically engineered 39006 *E. coli* and purified following a previously established protocol (Figure 1A). The purified GVs were observed by TEM, revealing the number of GVs was 25–58 (Figure 1B,C) in a single Serratia, while significantly more GVs were observed in a single *39006 E. coli*, about 35–290 (Figure 1B,C). Isolated GV_Ser_ displayed a consistent cylindrical shape, with about a 50 nm width and 250 nm length, but the engineered GV_E.coli_ exhibited slight variations in length, ranging from 50 nm to 400 nm (Figure 1B). The average particle sizes of GVs determined by Zetasizer were 164.53 ± 1.47 nm for GV_Ser_ and 147.5 ± 2.10 nm for GV_E.coli_ (Figure 1D,E), with PDIs of 0.12 ± 0.01 for GV_Ser_ and 0.12 ± 0.02 for GV_E.coli_. Furthermore, the zeta potentials of GVs were measured as −24.91 ± 0.51 mV for GV_Ser_ and −23.51 ± 1.16 mV for GV_E.coli_, respectively (Figure 1F). These results indicated that GV_E.coli_ and GV_Ser_ exhibit similar particle sizes and zeta potentials despite some differences in the particle size distribution observed under TEM.

### 2.2. In Vitro Ultrasound Imaging of GVs

**Figure 2:**
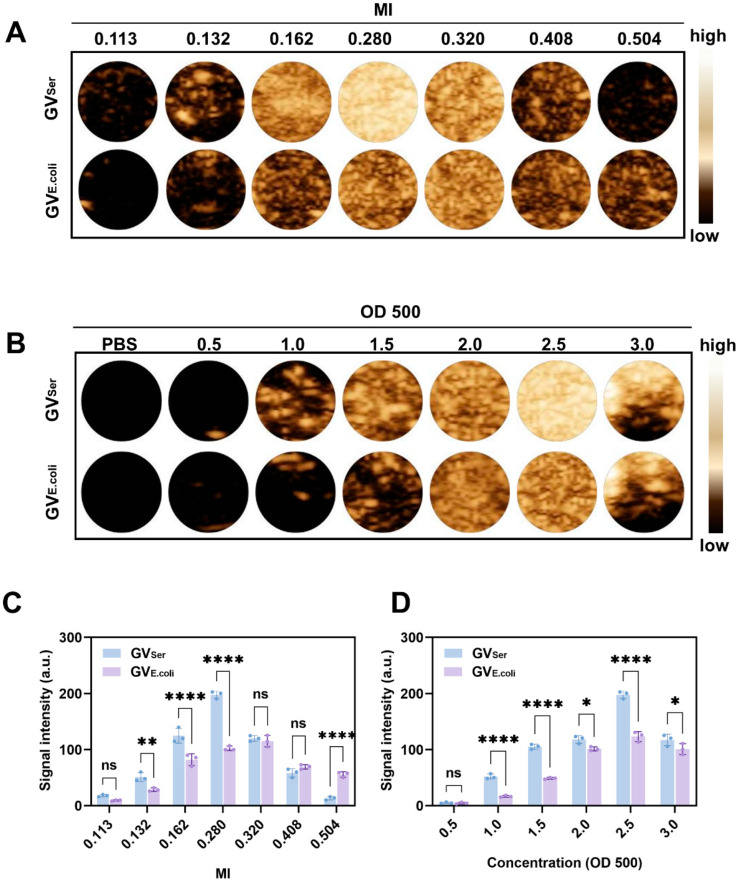
Ultrasound contrast imaging of GVs in vitro. (**A**) Nonlinear contrast images obtained using a linear array transducer of 200 μL GVs at OD500 2.5 when MI was changed from 0.113 to 0.504; (**B**) Nonlinear contrast images with different concentrations (OD500 0.5–3.0) of GVs; (**C**) Contrast signal intensity generated by GVs at different MIs; (**D**) Contrast signal intensity generated by GVs at different ODs. Statistical significance levels are denoted as **** for *p* < 0.0001, ** for *p* < 0.01, * for *p* < 0.05, and ns for no statistical significance.

Next, we assessed the ultrasound contrast imaging ability of GVs in agar phantoms using a clinical ultrasound machine equipped with linear array transducer at a frequency of 6.7 MHz. By maintaining the GV concentration at OD500 2.5, the contrast imaging characteristics were examined through ultrasound measurements at various MIs. Figure 2A,C revealed that the average signal intensity at the region of interest (ROI) of GV_Ser_ gradually enhanced with the increase of the MI, peaking at 197.8 ± 5.2 a.u. at MI = 0.280. However, the average signal intensity exhibited a decline until disappearance when the MI further increased over 0.280. By contrast, the average signal intensity of GV_E.coli_ displayed continuous enhancement from 0.113 to 0.320, reaching 115.9 ± 8.1a.u. at MI = 0.320 (Figure 2A,C). An MI of over 0.32 would result in the decay of ultrasound contrast signals. The possible explanation for this observation is the collapse of GVs under high acoustic power intensity, which exceeds the threshold MI of GVs. Subsequently, further evaluation of the influence of concentration on GV imaging was conducted. We found that with a concentration range of the OD500 from 0.5 to 3.0, the contrast signal exhibited an increase in intensity as the GV concentration rose from OD500 0.5 to 2.5. Notably, the acoustic wave experienced attenuation when the concentrations of the GVs were over OD500 2.5, leading to a diminished signal in the bottom part of the gel phantom (Figure 2B,D). Through in vitro imaging images and quantification, it can be seen that the CEUS effect of GV_Ser_ is significantly better than that of GV_E.coli_, which may be related to the uniformity of the particle size. Although the signal of genetically engineered bubbles is relatively weak, it is also observed that they have higher mechanical index tolerance, which is crucial for the stability and integrity of later preservation.

### 2.3. In Vivo Ultrasound Imaging of GVs

**Figure 3:**
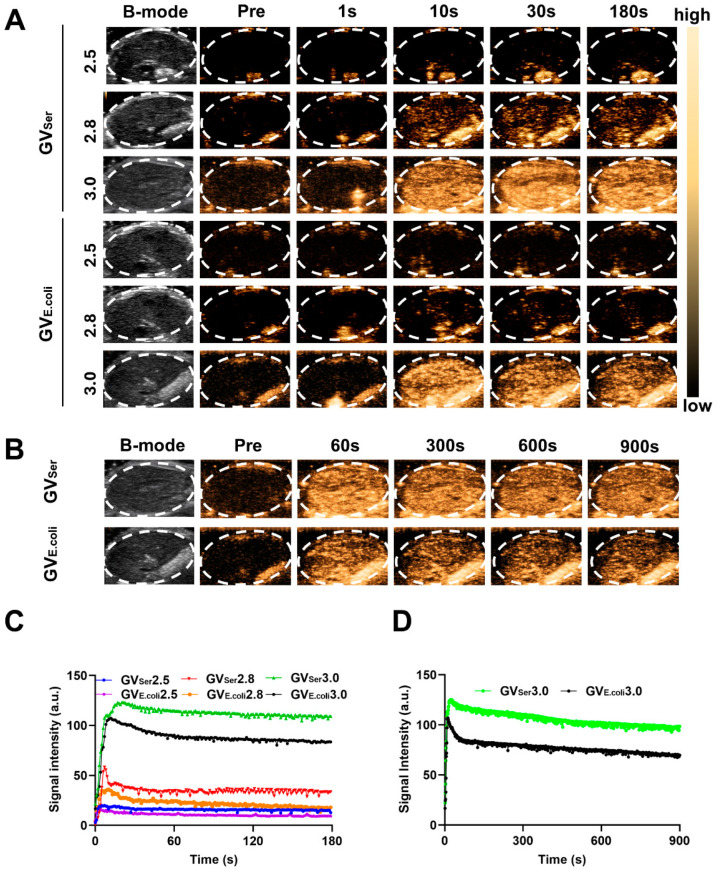
Ultrasound contrast imaging of GVs in vivo. (**A**) B-mode and nonlinear contrast images of GV_E.coli_ and GV_Ser_ (OD500 2.5, 2.8, 3.0) at different times after tail injection; (**B**) B-mode and nonlinear contrast images of GV_E.coli_ and GV_Ser_ at OD500 3.0; (**C**) Time–intensity curves of GVs at different concentrations in liver within 3 min; (**D**) Time–intensity curves of GVs at OD500 3.0 in liver within 15 min.

**Figure 4:**
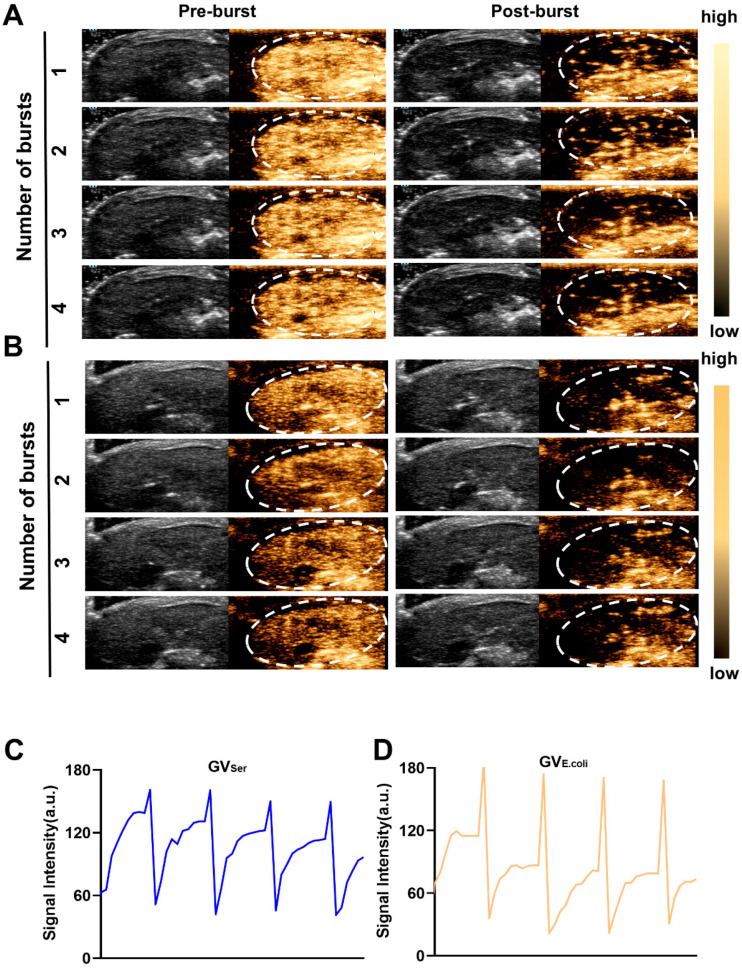
Re-perfusion of GVs after multiple ultrasonic bursts in vivo. (**A**,**B**) B-mode and nonlinear contrast images of GVs’ re-perfusion processes after four ultrasonic bursts, about 10 s between two bursts (A for GV_Ser_, B for GV_E.coli_); (**C**,**D**) Time–intensity curve of contrast signals of GVs during four ultrasonic bursts.

To further evaluate the imaging capability of GVs in vivo, various concentrations of GVs were administered via tail vein injection to C57BL/6 mice, followed by liver imaging in B-mode and contrast mode. At OD500s of 2.5 and 2.8, a relatively weak contrast signal was observed in the liver. Nonetheless, the contrast enhancement signals exhibited strong contrast signals at an OD500 of 3.0, achieving 76.0 ± 11.3 a.u. for GV_E.coli_ and 98.2 ± 9.9 a.u. for GV_Ser_, respectively. Both GV_E.coli_ and GV_Ser_ could maintain stable contrast signals for 15 min (Figure 3A–C). To further confirm the contrast signal from GVs, a short high-power burst (1 s) was utilized to disrupt the GVs. The contrast signals dramatically reduced after a high-energy burst but gradually reappeared in the liver. The disappearance and reappearance of contrast signals could be observed multiple times (at least four times) when the high-power bursts were repeated, as depicted in Figure 4A,B, confirming that the contrast signals really resulted from the GVs.

### 2.4. Imaging of Tumor by GVs

**Figure 5:**
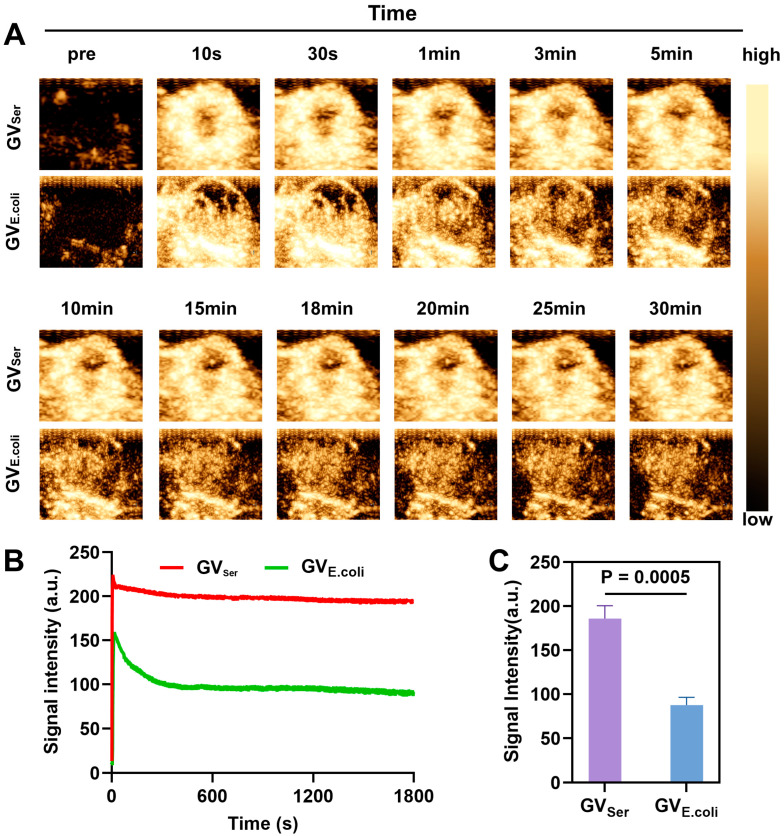
Ultrasound imaging of GV_Ser_ and GV_E.coli_ in tumor. (**A**) Nonlinear contrast images of GV_Ser_ and GV_E.coli_ 30 min after tail injection; (**B**) Time–intensity curve of GV_Ser_ and GV_E.coli_ perfused into the tumor tissue; (**C**) Contrast signal intensities of GV_Ser_ and GV_E.coli_ in the tumor at the 30th minute.

To examine the tumor imaging capability of GVs, we intravenously administered GV_Ser_ or GV_E.coli_ at equal concentrations into the tumor-bearing mice. From Figure 5A,B, we can see that both GV_Ser_ and GV_E.coli_ effectively perfused into the tumor. The imaging duration could last for at least 30 min and kept high-contrast signal intensities level (Figure 5B). This persistent and robust contrast enhancement within the tumor region indicated the efficient and thorough distribution of GVs, highlighting their potential as promising imaging agents for tumor detection and characterization. Notably, the contrast signal generated by genetically encoded GV_E.coli_ was weaker than that of native GV_Ser_, possibly due to the presence of a greater number of small GVs in GV_E.coli_ (Figure 5C).

### 2.5. In Vivo Toxicity Assessment

**Figure 6:**
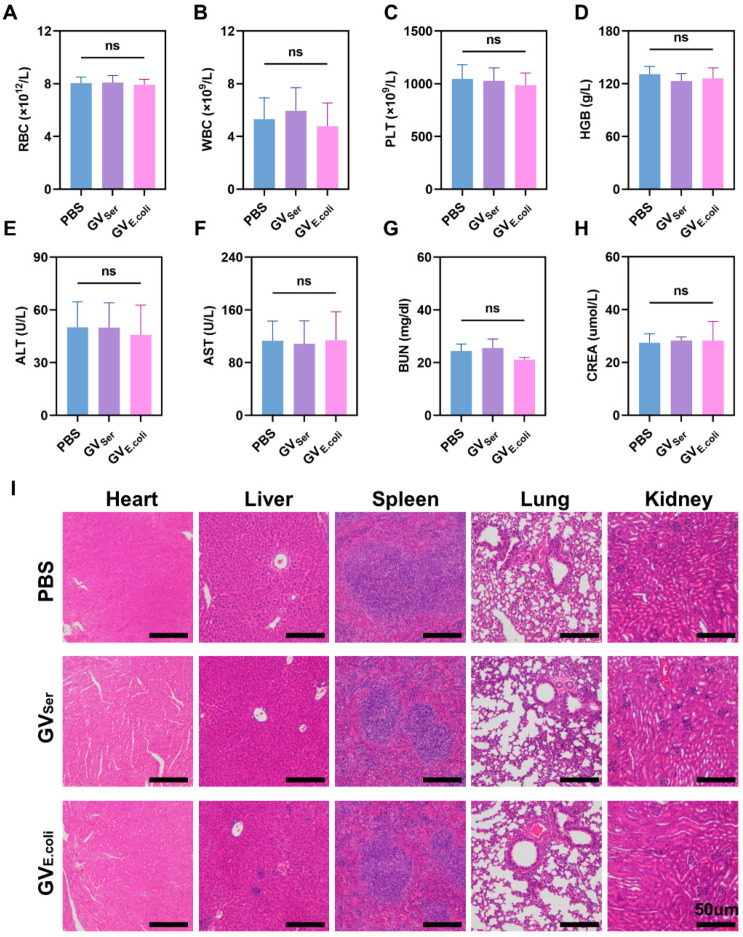
In vivo toxicity assessment. (**A**–**H**) The level of blood biochemical indicators, including RBC, WBC, PLT, and HGB; liver function (ALT, AST), and renal function (BUN, CREA) one day post-injection of PBS control or GVs (n = 4). (**I**) H&E staining of major organs (heart, liver, spleen, lung, and kidney) in mice with GV_Ser_, GV_E.coli_, and PBS at OD500 3.0. The scale is 50 µm. ns for no statistical significance.

To confirm the biosafety of these GVs in vivo, blood samples were collected from mice that were systemically injected with GV_Ser_, GV_E.coli_, or a PBS control. The levels of red blood cells (RBC), white blood cells (WBC), platelet count (PLT), and hemoglobin (HGB); indicators of liver function such as alanine aminotransferase (ALT) and aspartate aminotransferase (AST); and markers of renal function, including blood urea nitrogen (BUN) and serum creatinine (CREA), were detected, revealing all of them were within normal physiological ranges (Figure 6A–H). Furthermore, histological examination through H&E staining showed that there was not significant pathological damage in main organs such as the heart, liver, spleen, lungs, and kidneys of the mice treated with GV_Ser_ or GV_E.coli_ (Figure 6I). These findings provide the important evidence supporting the safety and biocompatibility of GVs, suggesting their clinical translation value as a promising tool for biomedical imaging and therapeutic applications.

## 3. Discussion

In recent years, the development of various nanoscale contrast agents has become a revolutionary process in the field of ultrasound contrast agents. In particular, gas vesicles (GVs) biosynthesized by aquatic bacteria, archaea, or algae have become a promising alternative to traditional chemically synthesized contrast agents [17]. In comparison with traditional chemically synthesized microbubbles, these biosynthetic GVs have many advantages. Firstly, a single GV usually exhibits a cylindrical or spindle-shaped protein nanostructure with a length range of about 100 nm to 600 nm and a width of about 45 nm to 200 nm [18], which has a significantly smaller particle size than microbubble contrast agents [9]. The GV wall is composed of 2 nm thick amphiphilic shells (the core structure is GvpA and GvpC), making GVs that have relatively high structural stability and controllability [5]. Secondly, the monodisperse particles of GVs ensure reliable and accurate imaging results and eliminate the variability. In addition, the gene-encoded protein shells of GVs allow them to be easily functionalized by genetic engineering procedures. For example, the tumor-targeting GVs can be designed at the DNA level by fusing the tumor-targeting peptides or antibodies to the Gvp C proteins, which are located on the surface of GVs. The development of bio-synthesized nanoscale ultrasound contrast agents represents a significant advancement, offering a promising avenue for biomedical applications [19,20,21].

In this study, we successfully isolated nanoscale GVs from wild-type Serratia and heterologously expressed the gas vesicle gene cluster in *E. coli*, leading to the production of nanoscale GVs. The average particle size of 100–200 nm enables them to penetrate blood vessels to reach adjacent tumor tissues, which is believed to be mainly due to the enhanced permeability and retention (EPR) effect [22]. The EPR effect, first proposed by Maeda et al., is recognized as a fundamental principle of cancer drug delivery and cancer nanomedicine design [23,24,25]. In our previous study, it was found that the biosynthesis of GVs from *Halobacterium NRC-1* lasted for a long time (15 min) after injection, while MB only lasted for less than 10 min [16], this may be related to the high permeability of blood vessels and poor lymphatic drainage in tumors [22]. In this experiment, we purified biosynthetic GVs, which can be injected intravenously for at least 30 min in tumors and can be maintained at a high level. This may be due to its unique physical properties of GVs, such as 100–200 nm size, negative charge, and rod-like biological characteristics. Nanoparticles with these characteristics are considered to be ideal drug-loading media for cancer treatment and have great potential to implement the EPR effect [26,27,28]. In the previous literature, nanobubbles with small particle size have always been a challenge in ultrasound contrast imaging, mainly due to their limited resolution, echo signal attenuation, and fast decay in the circulation [29]. In this study, we successfully used clinical equipment to achieve real-time imaging of small particle size GVs by optimizing imaging parameters. Although genetically engineered GVs show slightly weaker signals both in vitro and in vivo, which may be related to their size differences and long, rod-like morphology [30], the contrast signal intensity they exhibit is still sufficient to reflect their application potential. In particular, the multifunctional and highly adjustable characteristics of genetically engineered GVs provide great hope for a variety of applications, including molecular imaging, ultrasound-mediated drug delivery, and in vivo cell tracking and acoustic manipulation [31,32,33]. These applications of GVs in biomedicine can not only broaden its value in disease diagnosis and treatment but also deepen our understanding of the mechanisms of cell therapy [34,35]. Together, our study shows the immense potential of genetically engineered GVs for novel ultrasound contrast agents.

Of course, we are still faced with numerous challenges to apply newly developed biological nanobubbles in clinical settings. Firstly, to obtain GVs with stable uniform size and good dispersity remains a significant challenge, especially in the realm of genetic engineering. In order to overcome this problem, the process of genetic modification research, culture, and extraction of GVs still needs to be explored. Secondly, the stability of GVs poses a challenge due to the nanoscale size of the bubbles, especially during long-term storage. Although current methods involving low-temperature environments and stabilizers can extend their shelf life, further research is needed to consider more effective large-scale storage methods. Additionally, the biological compatibility of the bubbles is an important concern. While preliminary data suggest that the bubbles do not exhibit significant short-term side effects and demonstrate good tolerance to repeated injections, further research is necessary to study their long-term toxic effects, particularly on immune responses. Existing studies indicate that PEG modification can significantly reduce the immunogenic response of the bubbles [6]. However, we must remain cautious about their potential hazards. Despite these challenges, biological nanobubbles still hold vast prospects in the fields of ultrasound and biomedicine.

## 4. Materials and Methods

### 4.1. Materials

The *Serratia. 39006* strain used in this study was purchased from the American Type Culture Collection (ATCC). SoluLyse Bacterial Protein Extraction Reagent (Tris Buffer) was purchased from Shenzhen Chemical Test Technology Co., Ltd. (Item Number: L200500, Brand: Galantis, Shenzhen, China); Lysozyme (Item number: L8120-50g, Brand: Soleibao, Beijing, China) and DNase I (CAS Number: 9003-98-9, Brand: GLPBIO, Shanghai, China) were purchased from Beyotime Institute of Biotechnology. LB medium (no sugar) (one liter contains 10 g tryptone, 5 g yeast abstract, and 5 g NaCl) and LB agar (without sugar) (LB medium with agar 15 g/L) from HuanKai Microbial (Item number: 28324, Guangzhou, China) were used for E. coli strain cultivation. C57BL/6 mice were purchased from the Guangdong Provincial Center for Experimental Animals.

### 4.2. Isolation of Gas Vesicles

*Serratia. 39006* was cultured in LB medium, maintaining optimal conditions of 30 °C and a steady agitation of 200 rpm over a 72 h period. The cultured *Serratia. 39006* bacteria were collected through centrifugation at 800 *g* for 3 h. The collected bacterial samples were subsequently lysed with SoluLyse Bacterial Protein Extraction Reagent and lysozyme and gently stirred at room temperature for 2 h, followed by the addition of DNase I and incubation for another 2 h. The gas vesicles (GVs) were isolated through centrifugation at 800 *g*, 4 °C for another 3 h.

The gene encoding GVs was cloned from *Serratia. 39006* by PCR using Novizan P521 (Phanta SE Super-Fidelity DNA Polymerase) and assembled into a pET28a (+) vector (Novagen) via Gibson. Constructs were validated by Sanger sequencing, and primer details are provided in Supplementary Material Table S1. Plasmids encoding GVs were transformed into chemically competent *E. coli BL21(AI)* cells (Item number: C607003; Brand: Thermo Fisher Scientific; Guangzhou, China) and grown in 5 mL starter cultures in LB medium with 50 µg/mL kanamycin, 1% glucose for 16 h at 37 °C. Large-scale cultures in LB medium containing 50 µg/mL kanamycin and 0.2% glucose were inoculated at a ratio of 1:100 with the starter culture. When the optical density at 600 nm (OD 600) reached 0.6–0.7, 0.5% L-arabinose and 0.4 mM IPTG were added and the culture was further incubated at 30 °C for 22 h. After settling in a separating funnel for two days, the upper layer with bubble-containing bacteria was collected, and GVs were extracted using the same steps as for GV_Ser_. The purified GV_Ser_ and GV_E.coli_ were stored at 4 °C. The concentration of GVs within these preparations was accurately quantified using a spectrophotometer at a 500 nm wavelength.

### 4.3. Characterization of GVs

GV_Ser_ and GV_E.coli_ were diluted and carefully placed upon copper mesh. These specimens were then negatively stained using 2% phosphotungstic acid and subsequently left to dry at an ambient temperature. The morphologies of GVs were examined by a Transmission Electron Microscope (TEM, Hitachi H-7500 model provided by Hitachi Limited, Tokyo, Japan) and phase-contrast microscopy (Olympus IX83 inverted microscope, Tokyo, Japan). The particle size and zeta potential of GVs were measured using a Zetasizer analyzer (Zetasizer NanoS90, Malvern, Worcestershire, UK). All samples were diluted to the optimal concentrations under room temperature conditions. For each sample, the particle size and zeta potential were determined with three measurements.

### 4.4. In Vitro Ultrasound Imaging

The in vitro ultrasound imaging capability and mechanical index (MI) of GV_Ser_ and GV_E.coli_ were assessed at various concentrations using an ultrasound diagnostic device. To prepare the agar phantom, agar powder was mixed with distilled water, heated until clear, and then poured into molds with inserted eppendorf tubes. After cooling and setting, the eppendorf tubes were removed to create agar wells. GVs of different concentrations were added to the wells, and imaging was conducted using an ultrasound device with a linear array transducer (Mindray Resona 9T, Mindray, Shenzhen, China). The parameters were kept as follows: frequency: 7.1 MHz, depth: 2.5, frame rate: 10, dynamic range: 115, contrast gain 70 dB. To evaluate the impact of MI on GV imaging, the imaging performance of GVs was assessed at different MIs, ranging from 0.113 to 0.504, using GVs at OD5002.5. Based on the selected optimal imaging MI, further evaluation of the influence of concentration on GV imaging was conducted, with an OD500 concentration range of 0.5 to 3.0.

### 4.5. In Vivo Ultrasound Imaging

Animal experiments were conducted in accordance with protocols approved by the Ethics Committee of Shenzhen Institutes of Advanced Technology, Chinese Academy of Sciences. For liver imaging of GVs, male C57BL/6 mice were maintained under isoflurane anesthesia on a heating pad. GV_Ser_ and GV_E.coli_ with OD500 values of 2.5, 2.8, or 3.0 were intravenously injected into the mice and imaged using a line array transducer of Mindray Resona 9T in contrast mode. The MI was adjusted to the optimal imaging condition for each injection, and other parameters were set as follows: frequency: 7.1 MHz, depth: 2.5, frame rate: 10, dynamic range: 115, contrast gain 70 dB. A volume of 100 µL of GV was administered each time, and imaging was continuously performed for 3 min. To prevent GV aggregation in the liver, each mouse only received one injection a day, and the second injection was given at least one day later. When a GV at an OD500 3.0 was injected, a pulse was applied to burst the GVs 15 min post-injection. For tumor imaging of GVs, C57BL/6 male mice were subcutaneously injected with 2 × 10^5^ MB49 cells in 100 µL PBS. When the tumor diameter reached over 5 mm, 100 µL of GV with an OD500 of 3.5 was intravenously injected, and imaging was conducted using the same equipment. Images were continuously acquired for 30 min, and short high-power pulses were applied to burst these bubbles inside the tumor to prevent interference with the residual ultrasound contrast signals. A wait time of at least one day was allowed between two imaging sessions. Image processing and quantification were performed using Image J software (Image J/Fiji w11), and the time–intensity curve (TIC) was generated based on the average gray signal value at each time point.

### 4.6. Toxicity Assay

The in vivo biosafety of GV_Ser_ and GV_E.coli_ was evaluated by blood routine examination and histological analysis of major organs. In brief, 12 healthy C57BL/6 male mice were randomly divided into three groups: Group A received an injection of 100 µL PBS, Group B received an injection of 100 µL GV_Ser_, and Group C received an injection of 100 µL GV_E.coli_. Blood samples were collected 24 h after injection. Meanwhile, the major organs, such as the heart, liver, spleen, lung, and kidney, were removed for tissue sectioning and H&E staining (Wuhan servicebio technology Co., Ltd., Wuhan Sevier Biotechnology Co., Ltd., Mindray automatic veterinary Blood Analyzer, Model: BC-2800vet, Wuhan, China).

### 4.7. Statistical Analysis

The data were expressed as mean ± standard deviation. Independent *t*-tests were used for comparisons between two groups. One-way analysis of variance (ANOVA) followed by a Bonferroni multiple comparison test was used for comparisons among multiple groups. GraphPad Prism software (GraphPad Prism 9) was used for data visualization and statistical analysis. * *p* < 0.05 was considered statistically significant.

## 5. Conclusions

In this study, we successfully isolated nanoscale GVs from wild-type Serratia and further heterologously expressed the gas vesicles in *E. coli*. The in vitro and in vivo contrast imaging performance of GV_Ser_ and GV_E.coli_ were detected, revealing the excellent ultrasound contrast-enhanced signals. Specially, the contrast imaging parameters were optimized by using clinical ultrasound diagnostic equipment. Our study provides a kind of novel use of nanoscale ultrasound contrast agents with great clinical translation prospects.

## Data Availability

Data are contained within the article and Supplementary Materials.

## Supplementary Materials

The following supporting information can be downloaded at: https://www.mdpi.com/article/10.3390/ph17060755/s1, Table S1: Primers used in this study.
