# Supplementary figures and images for: Characterization and Comparison of Contrast Imaging Properties of Naturally Isolated and Heterologously Expressed Gas Vesicles
Source: Pharmaceuticals (Basel). 2024 Jun 7;17(6):755. doi: 10.3390/ph17060755 (PMC11207003; doi:10.3390/ph17060755)

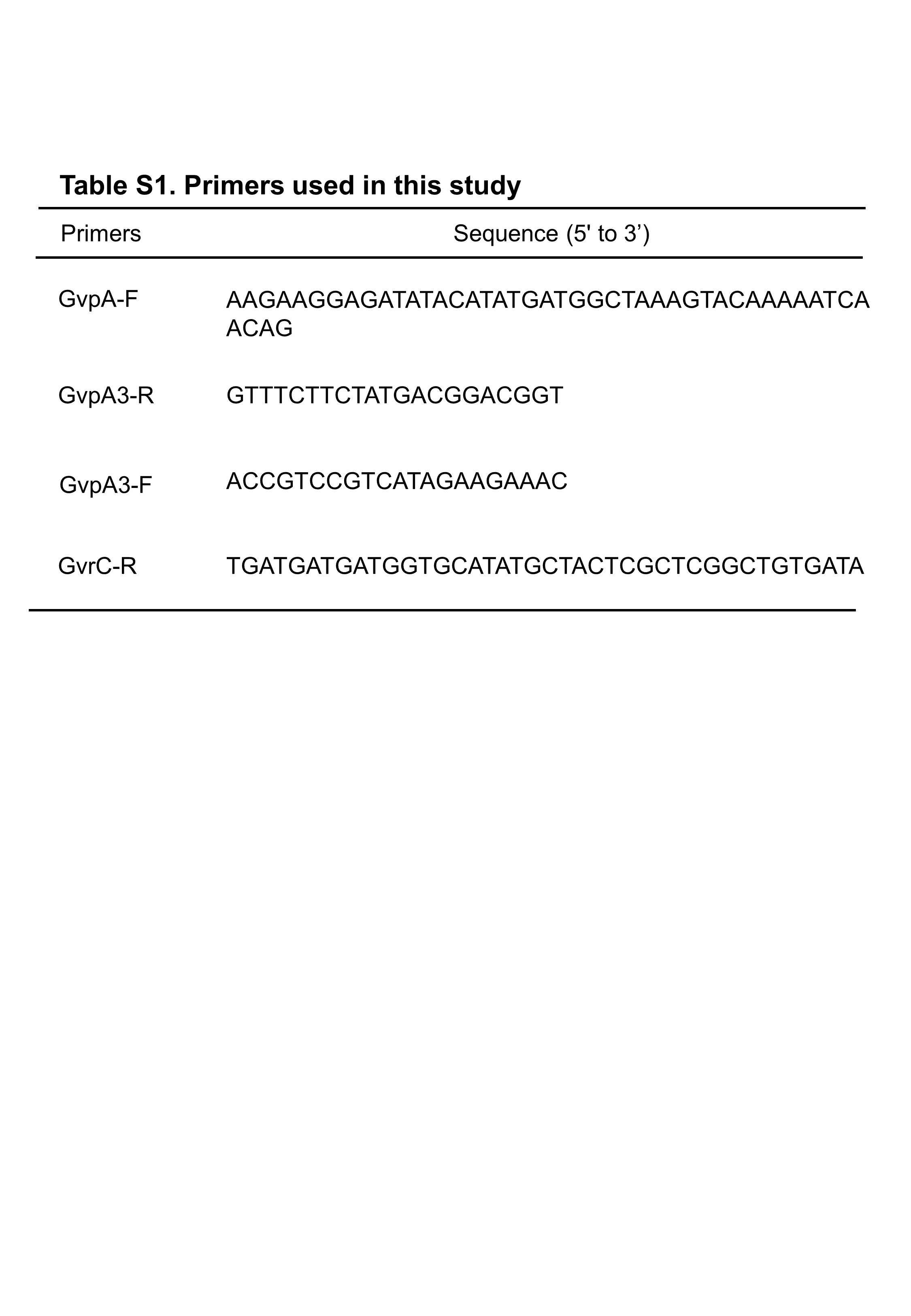

Supplement: Supplementary file 1 [file pharmaceuticals-17-00755-s001.zip › pharmaceuticals-3007374-supplementary.TIF]
